# Temperature-Dependent Resistive Properties of Vanadium Pentoxide/Vanadium Multi-Layer Thin Films for Microbolometer & Antenna-Coupled Microbolometer Applications

**DOI:** 10.3390/s19061320

**Published:** 2019-03-16

**Authors:** Mohamed Abdel-Rahman, Muhammad Zia, Mohammad Alduraibi

**Affiliations:** 1 Department of Electrical Engineering, College of Engineering, King Saud University, Riyadh 11421, Saudi Arabia; 2 KACST-TIC in Radio Frequency and Photonics for the e-Society (RFTONICS), College of Engineering, King Saud University, Riyadh 11421, Saudi Arabia; 3 Prince Sultan Advanced Technologies Research Institute (PSATRI), College of Engineering, King Saud University, Riyadh 11421, Saudi Arabia; fakhar.zia@psatri.org.sa; 4 Physics and Astronomy Department, College of Science, King Saud University, Riyadh 11451, Saudi Arabia; malduraibi@ksu.edu.sa; 5 National Center for Nanotechnology and Advanced Materials, King Abdulaziz City for Science and Technology (KACST), Riyadh 11442, Saudi Arabia

**Keywords:** microbolometer, temperature coefficient of resistance, vanadium oxide, multilayer structure, thermometer, temperature sensing, semiconductor, resistivity, antenna-coupled microbolometer

## Abstract

In this study, vanadium oxide (V_x_O_y_) semiconducting resistive thermometer thin films were developed, and their temperature-dependent resistive behavior was examined. Multilayers of 5-nm-thick vanadium pentoxide (V_2_O_5_) and 5-nm-thick vanadium (V) films were alternately sputter-deposited, at room temperature, to form 105-nm-thick V_x_O_y_ films, which were post-deposition annealed at 300 °C in O_2_ and N_2_ atmospheres for 30 and 40 min. The synthesized V_x_O_y_ thin films were then patterned into resistive thermometer structures, and their resistance versus temperature (*R*-*T*) characteristics were measured. Samples annealed in O_2_ achieved temperature coefficients of resistance (TCRs) of −3.0036 and −2.4964%/K at resistivity values of 0.01477 and 0.00819 Ω·cm, respectively. Samples annealed in N_2_ achieved TCRs of −3.18 and −1.1181%/K at resistivity values of 0.04718 and 0.002527 Ω·cm, respectively. The developed thermometer thin films had TCR/resistivity properties suitable for microbolometer and antenna-coupled microbolometer applications. The employed multilayer synthesis technique was shown to be effective in tuning the TCR/resistivity properties of the thin films by varying the annealing conditions.

## 1. Introduction

Microbolometers and antenna-coupled microbolometers have found applications in infrared, terahertz, and millimeter wave sensing [1,2,3,4,5,6,7,8,9,10,11,12]. Microbolometers are composed of several functional layers, namely: an absorber layer, a resistive thermometer layer, a thermal insulation layer, and a reflective layer. An absorber layer absorbs the incident long-wave infrared (LWIR) radiation (in the 8–12 µm band). The absorbed radiation heats the resistive thermometer layer causing a change in its resistance that is read by a dedicated readout integrated circuit (ROIC). In antenna-coupled microbolometers, the incident LWIR radiation is received by an antenna; the antenna resonant currents are then dissipated into an impedance-matched resistive thermometer layer causing a change in its resistance, which can be read by a dedicated ROIC. 

Two important parameters affect the performance of a microbolometer in both above-described configurations: the resistivity and the temperature coefficient of resistance (TCR) of the resistive thermometer layer. High TCRs lead to high responsivities. High TCRs are desirable to be accompanied with optimized resistivities suitable for the microbolometer resistance to be matchable to accompanying ROICs and coupled antennas (in antenna-coupled microbolometer configurations). The ability to tune the TCR/resistivity properties of the resistive thermometer layers is highlighted in this work through employing the multi-layer synthesis technique [13,14,15,16,17,18] toward developing V_x_O_y_ resistive thermometer thin films.

Semiconductors materials have been the preferred choice for the resistive thermometer layers in microbolometers due to their TCRs [1,2,4]. The resistance, *R*, of a semiconducting material changes with temperature, *T*, is in accordance with the Arrhenius relationship [4]:

(1) $$R(T)=R_{o}\cdot e^{\left(\frac{\Delta E}{k\cdot T}\right)}$$

where *R_o_* is a constant, Δ*E* is the activation energy, and *k* is Boltzmann’s constant. Accordingly, the TCR of a semiconducting material is given by [4]

(2) $$\mathrm{TCR}=\frac{1}{R}\frac{dR}{dT}=-\frac{\Delta E}{k\cdot T^{2}}.$$

High TCRs (typically between −2 and −5%/K) are achievable using semiconducting materials such as vanadium oxide [13,14,15,16,17,18,19,20,21,22], amorphous silicon [22,23,24,25,26], and other materials [26,27,28,29,30]. The high TCRs are accompanied with resistivity values (typically between 0.07 and 8000 Ω·cm) suitable for resistive thermometer layers in microbolometer configurations. The achieved resistivity values at high TCRs, however, cannot be matched to real antenna impedances (ranging typically between 50 and 300 Ω) in antenna-coupled microbolometer configurations. This fact obligated the use of low TCR metals as resistive thermometers in antenna-coupled microbolometer configurations [5,6,7,8,9,11,12], which is one of the main causes of the low responsivities in antenna-coupled microbolometers.

The multi-layer synthesis technique has been successful in realizing thermometer thin films with resistivities and TCRs suitable for microbolometer applications [13,14,15,16,17,18]. This synthesis technique relies on depositing cascaded alternating layers of a vanadium oxide (e.g., vanadium sesquioxide (V_2_O_3_) [18] or vanadium pentoxide (V_2_O_5_) [13,14,15,16,17]) and vanadium (V) metal and then annealing the structure to allow for intermixing between the vanadium oxide layers and the V metal layers, thus forming a mixed phase vanadium oxide, V_x_O_y_, [15] with desirable TCR and resistivity values [13,14,15,16,17,18]. The multi-layer synthesis technique allows one to tune the TCR and resistivity values of the thin films by changing the ratio between the thicknesses of the vanadium oxide layers and V metal layers and by varying the annealing conditions.

In this work, we apply the multi-layer synthesis technique toward fabricating resistive thermometer thin films with TCR and resistivity values specifically suitable for antenna-coupled microbolometer applications. To this end and by considering the findings of previous works [13,14,15,16,17,18], the vanadium/(vanadium oxide) ratio was increased in the deposited thin film, aiming for both a reduction in the resistivity of the synthesized thermometer thin film and for a minimal decrease in the TCR. In this work, multi-layer structures consisting of 5-nm-thick alternating multi-layers of V_2_O_5_ and V are sputter-deposited and further ex situ annealed for 30 min and 40 min in O_2_ and N_2_ atmospheres. Resistive thermometers were then fabricated, and resistance versus temperature (*R*-*T*) characteristics were measured and analyzed.

## 2. Materials and Methods

**Figure 1:**
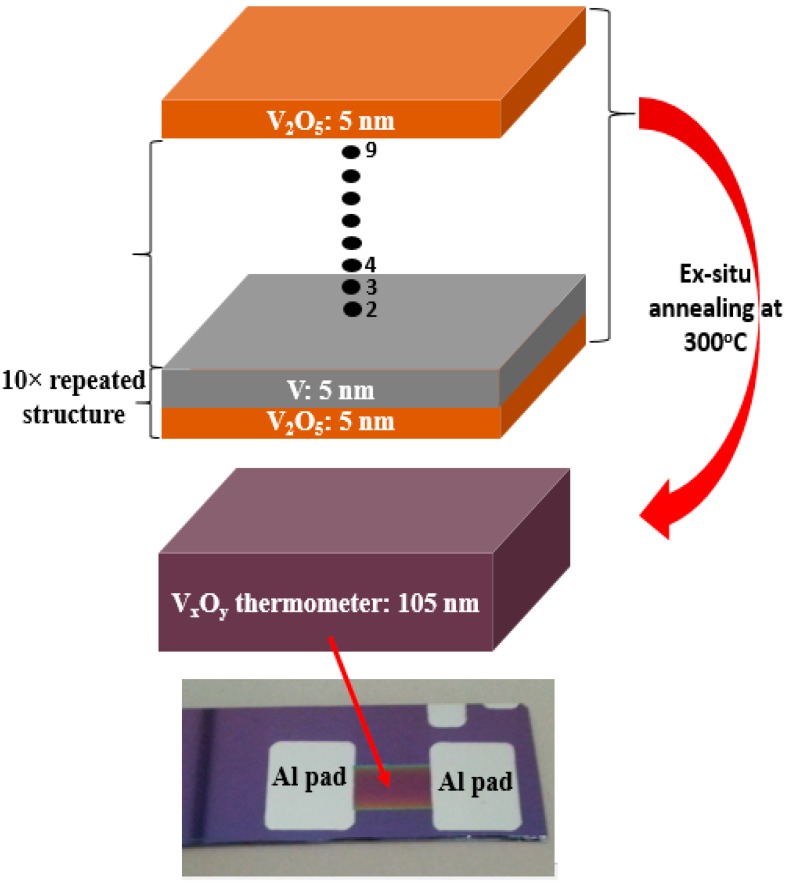
A schematic diagram of the synthesis process of the V_x_O_y_ thin films. A photograph of the fabricated resistive thermometer (bottom).

The synthesis process of the developed vanadium oxide, V_x_O_y_, thin films and a photograph of one of the fabricated resistive thermometers are shown in Figure 1. The V_x_O_y_ thin films were sputter-deposited on silicon substrates having 300 nm of silicon dioxide on top of them. The V_x_O_y_ thin films were synthesized via sputter coating alternating multi-layers of V_2_O_5_ and V, each having a thickness of 5 nm. The thickness of the whole sputter-deposited multi-layer structure is 105 nm. The deposited multi-layer structure was sputtered through a mechanical mask to yield a resistive thermometer with dimensions 9 mm × 6 mm. The V_2_O_5_ layers were sputter-deposited from a 99.5% pure V_2_O_5_ target at 150 W of RF power with a deposition rate of 0.75 nm/min. The V layers were sputter-deposited from a 99.9% pure V target at 150 W of DC power with a deposition rate of 4 nm/min. The sputtered multi-layered structures were then ex-situ annealed in a horizontal tube furnace at 573 K for 30 and 40 min in O_2_ and N_2_ atmospheres. Aluminum (Al) metal, having a thickness of 120 nm, was then DC sputter-deposited through a second mechanical mask to form the contact pads for the resistive thermometers. All sputter deposition operations were performed at a chamber base pressure of 2 × 10^−6^ Torr and at 3 mTorr of argon pressure.

For the purpose of electrical characterization, *R*-*T* measurements were performed. The measured results were then plotted and analyzed. The resistive thermometers were placed on a hot plate, which was in turn placed on the Cascade Microtech’s probe station. The hot plate’s temperature varied from 294.3 to 337 K, and the resistance of the thermometer structures was recorded in steps of approximately 5 K. For measuring the resistance, Agilent’s U1521 Multimeter was connected to the Mircotech’s microprobes, and the probes were made to contact the Al pads of the resistive thermometers.

## 3. Results

**Figure 2:**
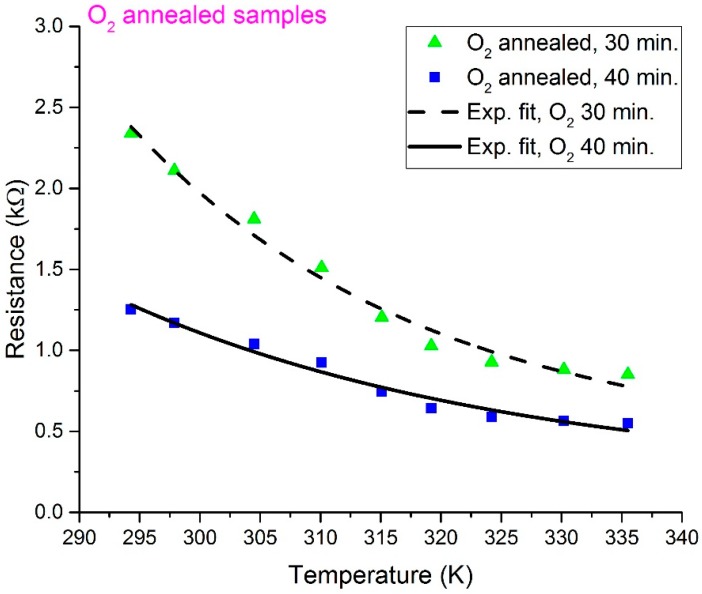
Measured resistance versus temperature for O_2_-annealed resistive thermometers.

**Figure 3:**
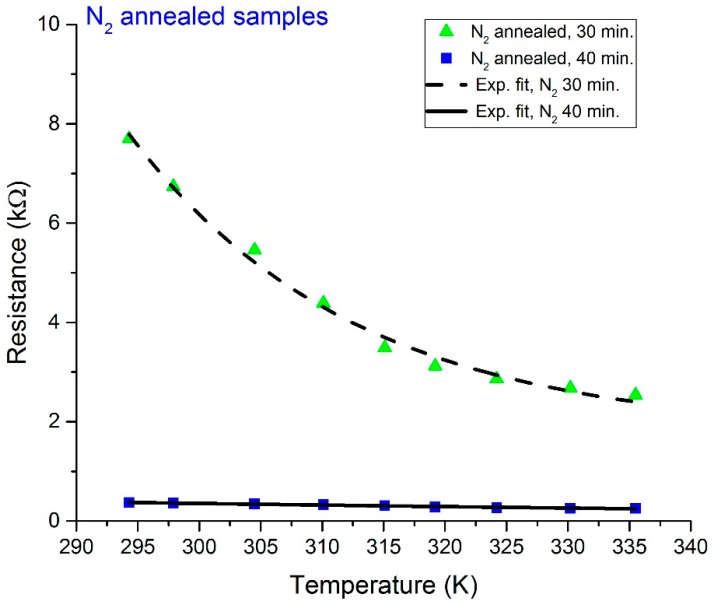
Measured resistance versus temperature for N_2_-annealed resistive thermometers.

**Figure 4:**
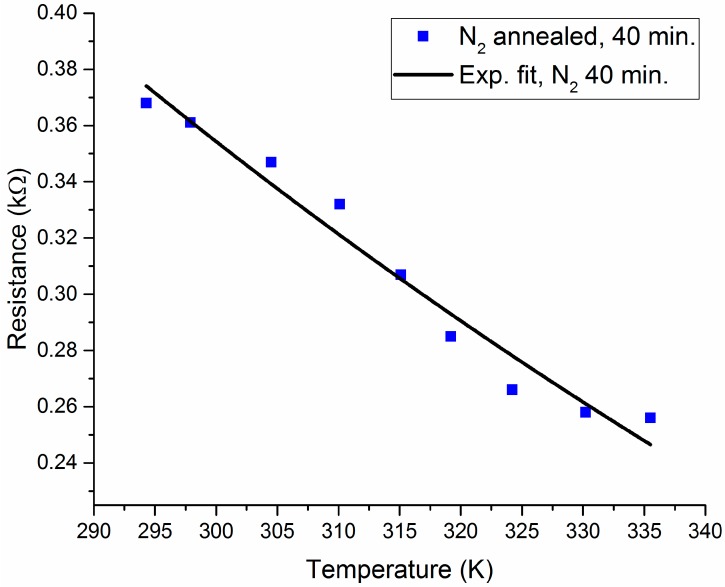
Measured resistance versus temperature for the resistive thermometers annealed in N_2_ for 40 min.

**Figure 5:**
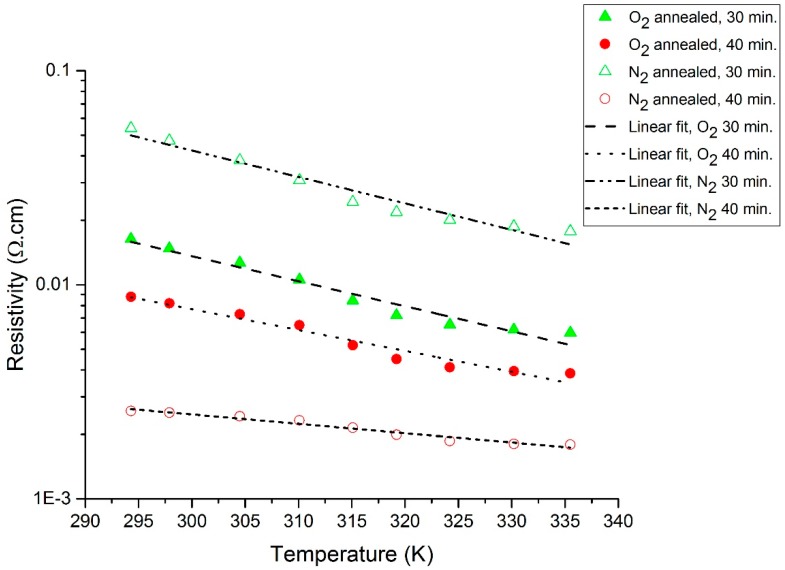
Resistivity (log scale) versus temperature plot for O_2_- and N_2_-annealed resistive thermometers.

**Figure 6:**
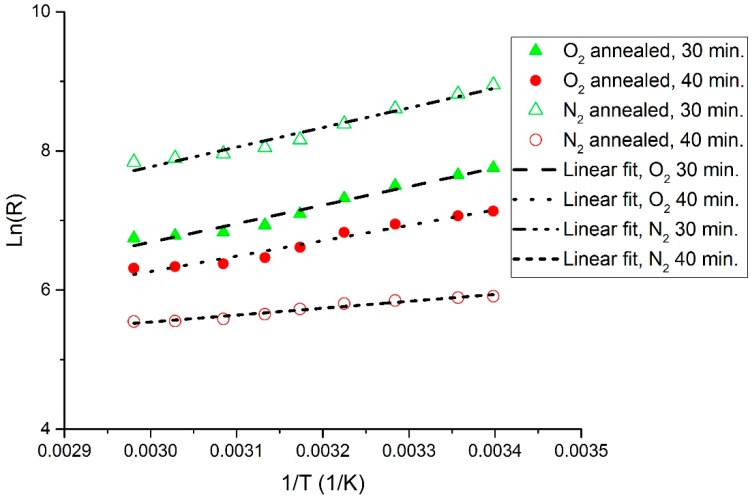
Ln(*R*) versus 1/*T* Arrhenius plot for O_2_- and N_2_-annealed resistive thermometers.

**Figure 7:**
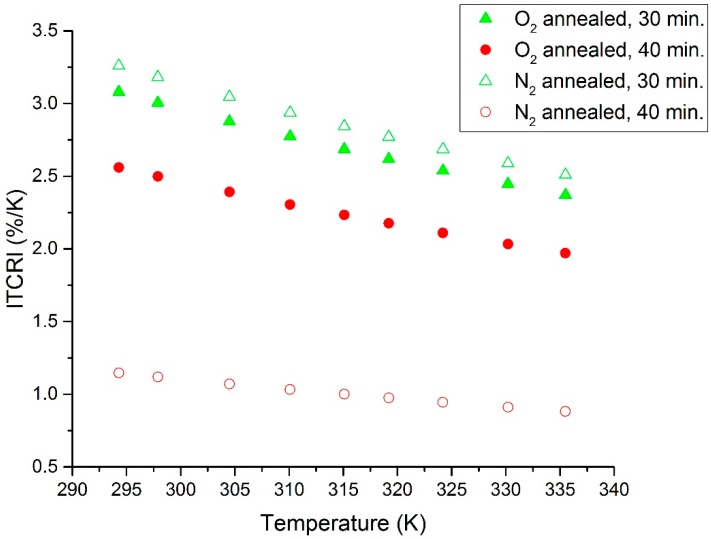
|TCR| versus temperature plot for O_2_- and N_2_-annealed resistive thermometers.

The measured *R*-*T* characteristic plots for the O_2_- and N_2_-annealed V_x_O_y_ thermometer thin films are shown in Figure 2 and Figure 3, respectively. In addition, Figure 4 displays a re-plot of the measured *R*-*T* characteristics for the structure annealed for 40 min. in N_2_; this figure is presented for better scale clarity. The relationship between resistance and temperature reveals a clear semiconducting behavior, as the resistance decreases with the increase in temperature. The resistance versus temperature behavior is commensurate with the Arrhenius relationship, and the measured curves were found to be best fit using an exponential decay relationship. The resistivity versus temperature relationships are plotted in Figure 5, and Ln (*R*) versus 1/*T* Arrhenius relationships are plotted in Figure 6. The activation energies were extracted from the slopes of the fitted curves. The TCRs were calculated using Equation (2). Furthermore, the |TCR| versus temperature plots are depicted in Figure 7. A summary of all the measured and extracted parameters is given in Table 1.

The measured resistance values (at ~298 K) for the resistive thermometers annealed in O_2_ atmosphere were 2.11 and 1.17 kΩ for the 30 and 40 min annealed samples, respectively. This corresponds to resistivity values of 0.01477 and 0.00819 Ω·cm. The corresponding TCRs are −3.0036 and −2.4964%/K, while the measured resistance values (at ~298 K) for the resistive thermometers annealed in the N_2_ atmosphere were 6.74 and 0.361 kΩ for the 30 and 40 min annealed samples, respectively. This corresponds to resistivity values of 0.04718 and 0.002527 Ω·cm. The corresponding TCRs are −3.18 and −1.1181%/K. The highest observed TCR (at ~294.3 K) was −3.26%/K for the 30 min annealed sample in the N_2_ atmosphere. The room temperature resistivity decreased as the annealing time increased for both the O_2_ and N_2_ annealing atmospheres. This might be due to the improved intermixing between the V and V_2_O_5_ layers. Annealing for a longer period results in a vanadium-rich thin film in which the diffused vanadium atoms increase its conductivity. The improved intermixing at longer annealing times can be further verified by examining the *R_o_* constant in Equation (1). The *R_o_* constant is related to the conductivity prefactor, which depends on the carrier mobility and the density of states at the conduction band edge [23]. The *R_o_* constant was extracted, and this is shown in Table 1. The *R_o_* constant was found to increase with annealing time for both O_2_- and N_2_-annealed samples, which may indicate a reduction in carrier mobility due to an increase in carrier concentration because of improved intermixing. Moreover, it was observed that the N_2_-annealed thermometer thin films show much higher change in resistivity as compared to the O_2_-annealed films. This was expected to occur during the 30 min of annealing in O_2_ due to the formation of an oxide phase, which was due to the presence of O_2_ in the furnace. This oxide phase caused the 30 min annealed sample to possess lower resistivity. This oxide phase did not form during the 30 min of annealing in N_2_ due to the absence of O_2_ in the furnace and the incomplete diffusion of V atoms. Increasing the annealing time to 40 min increased the diffusion of V atoms, causing a phase change due to the intermixing between the V/V_2_O_5_ layers, despite the absence of O_2_ during the N_2_ annealing. Our previous analysis in [15] proved that the thin films prepared by the multilayer synthesis technique are composed of a mixture of different vanadium oxide phases. The composing vanadium oxide phases and their composition ratios can assist in explaining the above-measured behaviors.

Furthermore, the work presented in this study was compared to other research studies [13,14,15,16,17,18] involving V_x_O_y_ thin films synthesized by sputter deposition of alternating multilayers of V/V_2_O_5_ or V/V_2_O_3_ followed by ex situ annealing at 300 °C in N_2_ or O_2_ atmospheres. By considering all the studies, it can be generally deduced that, increasing V content in V/V_2_O_5_ or V/V_2_O_3_ multilayer stacks reduces the resistivity of the synthesized thin film. When considering the studies concerned with the V/V_2_O_5_ multilayer stacks annealed at 300 °C in an N_2_ atmosphere, it can be seen that, for a V/V_2_O_5_ ratio of 5 [13], the resistivity value was found to be 3.6 Ω·cm at a TCR of −2.42%/K. For a V/V_2_O_5_ ratio of 3 [16], the resistivity value was found to be 2.68 Ω·cm at a TCR of −3.55%/K. For a V/V_2_O_5_ ratio of 1 (this work), the resistivity value was found to be 0.04718 Ω·cm at a TCR of −3.18%/K. Resistivity values are clearly decreasing with the increase in V content. Additionally, in alternating multilayers of V/V_2_O_5_, the resistivities and the TCRs of the thin films were both found to decrease as the annealing time increased from 30 to 40 min ([13], this work). Moreover, it can be generally deduced that thin films synthesized from V/V_2_O_3_ multilayer stacks [17,18] possess higher TCRs at any given resistivity value than thin films synthesized from V/V_2_O_5_ multilayer stacks [13,14,15,16]; a TCR of −3.72%/K at 0.72 Ω·cm was measured for a 30 min annealed sample in an N_2_ atmosphere [18].

## 4. Discussion

**Figure 8:**
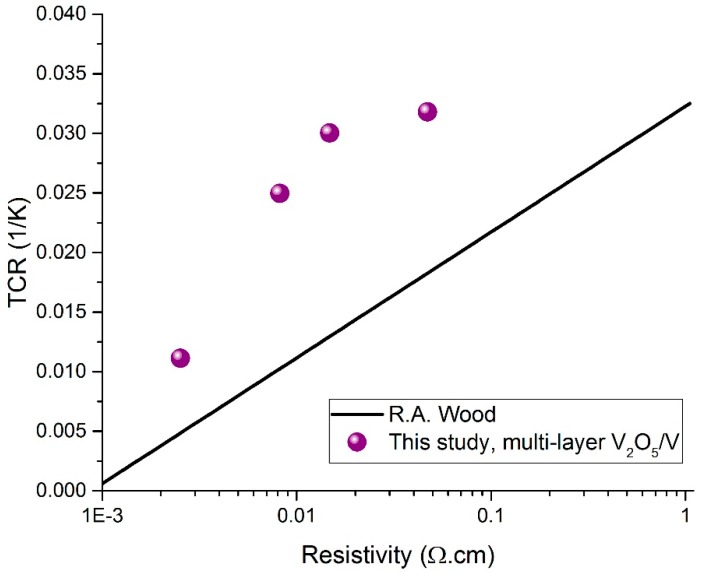
TCR versus resistivity for thermometer thin films developed in this work in comparison to the results published in [4].

The resistive thermometer thin films developed in this work exhibit relatively high TCR values at low resistivities values. This allows for employing such thin films in different microbolometer applications. Low resistivity yields low thermal noise in addition to low power dissipation in the microbolometer. This can be useful, especially, for microbolometers employed in low power thermal imaging systems where low power consumption is sought. It is generally known that the temperature sensing layers in commercial VO_x_ microbolometers have TCRs ranging from approximately −2 to −3%/K [1,2,4] with corresponding resistivities between 0.07 to 1.1 Ω·cm [1,2]. The resistive thermometer layers developed in this work possessing TCRs between −2 to −3%/K have lower resistivities, between 0.00819 and 0.04718 Ω·cm. Further, the TCR/resistivity performance of the resistive thermometers developed in this work were compared to the literature-standard results in [4], where VO_x_ thin films were produced using ion-beam deposition. This comparison is represented graphically in Figure 8. The line plot (black) represents the results in [4] and was generated by plotting the equation: TCR = 0.03227 + 0.010556·log(x), where x is the resistivity in Ω·cm. The violet dots represent the values achieved in this paper. It is clear from the plot that the resistive thermometer thin films developed in this work possess higher TCRs at any given resistivity value. Additionally, when comparing the results achieved in this work to the results in [31,32], where VO_x_ thin films were produced by reactive pulsed-dc magnetron sputtering with and without substrate bias, it can be seen that, in most of the cases, the work presented in this paper shows higher TCRs at any given resistivity value. Another notable result is the low resistivity, 2.527 × 10^−3^ Ω·cm, and moderate TCR, −1.1181%/K, achieved by annealing in an N_2_ atmosphere for 40 min. This resistivity yields thermometer resistances matchable to real antenna impedances in antenna-coupled microbolometer configurations. Here, it is worth pointing out the work in [10], where VO_x_ thermometers were coupled with gold (Au) antennas and yielded 5× higher infrared responsivity compared to Au antennas coupled with niobium (Nb) thermometers. The authors, however, revealed neither the synthesis method nor the resistance or TCR of the fabricated VO_x_ thermometer. Moreover, it is worth mentioning that the synthesis technique used in this work is CMOS-compatible, as the annealing temperatures do not exceed 300 °C; this makes the resistive thermometer layers suitable for monolithic integration with CMOS ROICs. Furthermore, for an estimate of the 1/*f* noise of microbolometers containing the thin films presented in this study, we referred to [33]. In [33], the noise of microbolometers based on V_x_O_y_ thin films fabricated using the multilayer synthesis method was found to be 29.41 μV/√Hz at a modulation frequency of 100 Hz; those devices were 1/*f* noise-limited. Accordingly, the 1/*f* noise of microbolometers containing the thin films presented in this study are expected to have 1/*f* noise values of the same order of magnitude.

## 5. Conclusions

In this paper, the multi-layer synthesis technique was used to realize resistive thermometers with different TCR & resistivity values useful for both microbolometer and antenna-coupled microbolometer configurations. When compared with other results, it can be seen that the TCR values achieved in this work are higher than others at any given resistivity value. This would be useful in making microbolometers in thermal imaging systems requiring low power consumption. Remarkably, the 40 min annealed thin film yielded a resistive thermometer with low resistivity, 2.527 × 10^−3^ Ω·cm, which allows matching a microbolometer to real antenna impedances in antenna-coupled microbolometer configurations and simultaneously maintain a moderate TCR value that allows for acceptable responsivity. The achieved results in this paper are promising and therefore suggest that more studies be done. Specifically, the 1/*f* noise performance and the compositional nature of the synthesized V_x_O_y_ thin films need to be studied.

## Figures and Tables

**Table 1:** Summary of measured and extracted electrical properties for V/V_2_O_5_ thin film resistive thermometers.

Sample ID	Resistance (kΩ) at R.T.	Resistivity (Ω·cm) at R.T.	TCR (%/K)	Δ*E* (eV)	*R_o_* (Ω)
O_2__30min.anneal	2.11	0.01477	−3.0036	0.229	0.162
O_2__40min.anneal	1.17	0.00819	−2.4964	0.191	41.797
N_2__30min.anneal	6.74	0.04718	−3.18	0.243	0.517
N_2__40min.anneal	0.361	0.002527	−1.1181	0.085	12.896
